# Researchers’ views on, and experiences with, the requirement to obtain informed consent in research involving human participants: a qualitative study

**DOI:** 10.1186/s12910-020-00538-7

**Published:** 2020-10-02

**Authors:** Antonia Xu, Melissa Therese Baysari, Sophie Lena Stocker, Liang Joo Leow, Richard Osborne Day, Jane Ellen Carland

**Affiliations:** 1 School of Medical Sciences, University of NSW, Sydney, NSW Australia; 2 Department of Clinical Pharmacology and Toxicology, St Vincent’s Hospital, Darlinghurst, NSW Australia; 3 Discipline of Biomedical Informatics and Digital Health, School of Medical Sciences, Faculty of Medicine and Health, The University of Sydney, Sydney, NSW Australia; 4 St Vincent’s Clinical School, University of NSW, Sydney, NSW Australia; 5 School of Medicine, University of Notre Dame Australia, Sydney, Australia

**Keywords:** Informed consent, Researchers’ views, Ethics, National Statement

## Abstract

**Background:**

Informed consent is often cited as the “cornerstone” of research ethics. Its intent is that participants enter research voluntarily, with an understanding of what their participation entails. Despite agreement on the necessity to obtain informed consent in research, opinions vary on the threshold of disclosure necessary and the best method to obtain consent. We aimed to investigate Australian researchers’ views on, and their experiences with, obtaining informed consent.

**Methods:**

Semi-structured interviews were conducted with 23 researchers from NSW institutions, working in various fields of research. Interviews were analysed and coded to identify themes.

**Results:**

Researchers reported that consent involved information disclosure, understanding and a voluntary decision. They emphasised the variability of consent interactions, which were dependent on potential participants’ abilities and interests, study complexity and context. All researchers reported providing written information to potential participants, yet questioned the readability and utility of this information. The majority reported using signed consent forms to ‘operationalise’ consent and reported little awareness of, and lack of support in implementing more dynamic informed consent procedures, such as verbal informed consent, that was fit for the purposes of their studies. Views on Human Research Ethics Committees (HRECs) varied. Some reported inconsistent, arduous inputs on the information form and consent process. Others expressed reliance on HRECs for guidance, viewing them as institutional safeguards.

**Conclusions:**

This study highlights the importance of transparent relationships, both between researchers and participants, and between researchers and HRECs. Where the relationship with study participants was reported as more robust, researchers felt that they were better able to ensure participants made better, more informed decisions. Where the relationship with HRECs was reported as more robust, researchers were more likely to view them as institutional safeguards, rather than as bureaucratic hindrances. Conscientious and mindful researchers are paramount to ensuring the procedure accommodates individual requirements. This study advocates that when designing ethical informed consent practices, researchers should be integrated as autonomous players with a positive input on the process, rather than, in the worst case, predatory recruiters to be curtailed by information forms and oversight.

## Introduction

Informed consent, in the context of research, is described as a ‘voluntary choice … based on sufficient information and adequate understanding of both the proposed research and the implications of participating in it’ [1]. It is often cited as the ‘cornerstone of research ethics’, [2] and the act of obtaining informed consent is seen as fundamental in satisfying ethical research principles of respect, beneficence and justice [3]. Despite its significance, attempting to derive a consensus statement on what constitutes a gold standard in obtaining informed consent is a fraught task. There is little consensus regarding the threshold of sufficient understanding for consent to be valid [4, 5], or the ideal way to obtain informed consent [6, 7]. Work exploring informed consent typically focuses on the need to improve participant understanding [8, 9]. Though important, little work has been undertaken in Australia within a domestic context, to investigate the perspective of researchers, despite the fact that they are party to the informed consent process and are responsible for initiating the informed consent interaction.

The requirements surrounding the obligation to obtain informed consent from research participants are also now much more complicated than when they were first articulated in the Nuremberg Code (1946) and the Declaration of Helsinki (1964). In most developed nations, regulation and accountability surrounding the requirement falls under the purview of government associated regulatory bodies. The National Institutes of Health in the United States (US), the Health Research Authority in the United Kingdom (UK) and the National Health and Medical Research Council (NHMRC) in Australia issue guidelines that require research projects be ethically approved by an independent review board before they can commence. In Australia, the principal guideline delineating the responsibility to obtain informed consent is the NHMRC’s National Statement on Ethical Conduct in Human Research (2007) (‘National Statement’). Despite the significance of the National Statement, studies show that researchers have varying degrees of familiarity with the document, ranging from ignorance to complete understanding [10, 11]. This variation in knowledge aligns with research findings that researchers recall very little of the training that they received on ethical issues relating to human participants in research [12].

Given the possibility that researchers may have limited recall or understanding of formal guidelines, questions arise relating to which principles they use to inform their practice. Normative principles relating to how consent *ought* to be obtained raise some important and difficult ethical questions, which have been the subject of rigorous academic debate. How much information should participants be required to understand before their decision to participate is valid [4, 5, 13, 14]? Is adequate understanding even possible, if, by definition, the outcomes of a research study are unknown [15]? Should participants’ trust in a researcher erode their capacity to make a voluntary decision [16]? There are currently few satisfactory answers.

Studies that seek to describe ‘gold standard’ practices for obtaining informed consent have led to the quantification of certain parameters of the informed consent process to assess its quality. These include a meta-analysis describing the correlation between information disclosure and understanding [6], studies quantifying the ‘readability’ of consent forms [13, 17], and interventional studies testing the efficacy of novel information delivery methods, including interactive multimedia aids [18]. However, while these studies are useful in describing phenomena, their utility in directing principles for improving practice is limited. Some studies declare that quantitative methodologies are unable to account for the influence of values such as trust and honesty, which will invariably affect the ethical issues in particular cases [8]. Informed consent cannot be viewed as simply a mechanical information disclosure. The relationship between two highly variable human beings is at the core of the practice.

A major element of informed consent stipulated by guidelines is that it needs to be a voluntary decision [1]. Guidelines warn against dependent relationships between researchers and participants, as this may erode a participant’s voluntary consent [1]. In particular, the role of the clinician-researcher has been criticised as being problematic, as it may cause conflicts of interest [9, 19, 20]. Despite this, studies show that researchers view the ethical concepts in research as similar to those in clinical care, and that experience in one sphere may improve practice in another [10]. Clinician-researchers in oncology have reported awareness that conflicts of interest may arise when enrolling their own patients, however they have also reported that an ongoing relationship was valuable in advising patients on the appropriateness of study participation [16]. Currently the views of Australian researchers on this topic are relatively unknown, including the role of a relationship between researcher and participant, and how the voluntary nature of a potential participant’s decision is protected.

While studies exploring the perspectives of researchers on the informed consent process have been undertaken, the majority of these have focussed on the understanding and difficulties that arise when obtaining informed consent in research studies with an international focus. While the outcomes of this work are important, the issues unveiled, such as the ethics of researchers from developed countries entering developing countries to recruit participants, remuneration, and language and cultural barriers to adequate informed consent, are less relevant to domestic research. A significant amount of research is undertaken in a domestic context, with domestic recruitment pools. Informed consent is no less important in these contexts, and the experiences and views of researchers in this context are poorly understood.

The present study aimed to explore Australian researchers’ views of, and experiences with, the requirement to obtain informed consent in domestic research.

The study had two primary research questions:

1. What are researcher’s views on, and understandings of, their obligation to obtain informed consent?
2. Where do researchers learn how to obtain informed consent, and is there sufficient support and guidance?

## Methods

Methods in grounded theory [21, 22] were adapted in this inductive qualitative study [23, 24]. An interpretivist epistemology was used, as we understood that multiple, equally valid, viewpoints about informed consent may exist [25–27].

Ethics approval was granted for this study by the Human Research Ethics Committee (HREC) of St Vincent’s Hospital, Sydney (LNR/18/SVH/143).

### Study participants

Snowball sampling [28] was initially used to enrol researchers (*n* = 23, Table 1) who worked with human participants across research institutes, hospitals and universities in metropolitan NSW. Researchers known to the research team were contacted by e-mail. Invitees were encouraged to refer colleagues to the study. Purposive sampling was used concurrently with analysis to capture a heterogeneous sample of research fields and study types [29]. Participation was voluntary and participants did not receive compensation.

**Table 1:** Interview participant demographics

	Number (%)
Total participants	23 (100)
Gender	
**Male**	8 (35)
**Female**	15 (65)
Experience obtaining informed consent (years)	
**0–5**	5 (22)
**5–10**	4 (17)
**> 10**	14 (61)
Affiliated institution^a^	
**Hospital**	11 (48)
**Research Institute**	8 (35)
**University**	14 (61)
HREC member^b^	
**Yes**	3 (13)
Highest level of education	
**Bachelors**	2 (9)
**Graduate Diploma/Masters**	10 (43)
**PhD and above**	11 (48)

^a^Percentages > 100%, as some researchers were affiliated with more than one institute

^b^Membership of Human Research Ethics Committees (HRECs) was not explicitly asked, only researchers who mentioned in passing that they were members of HRECs were noted

Broadly, research methods employed by researchers interviewed ranged from surveys and interviews to randomised controlled trials testing procedural and drug interventions. Some worked with ‘vulnerable participants’, including children and young people, people highly dependent on medical care and those with cognitive impairments. These categories are defined in Section 4 of the National Statement. Researchers worked in fields including psychology, medical sciences, and health services research and safety. Project descriptions were omitted to protect the privacy of participants.

### Interviews

A semi-structured interview guide was developed by a senior clinician researcher (RD), senior hospital scientists (JC, SS), a qualitative research expert (MB) and a research student (AX) and refined on the basis of pilot interviews. Topics included researchers’ views on informed consent, training and guidelines, the consent form, and difficulties faced in obtaining informed consent. (Supplementary file 1).

Interviews were conducted in person, one-on-one by AX, and lasted from 21 min to 59 min (mean = 38 min). Participants provided written informed consent for their interviews to be audio-recorded and for data to be used in this study.

### Analysis

Interviews were transcribed verbatim (AX). Non-identifiable transcripts were then analysed by two study investigators by applying inductive thematic analysis on the transcripts [23]. Transcripts were first read to gain meaning, and emergent themes were identified and then coded. Comparative analysis was also conducted across interviews to reaffirm or adjust subsequent coding. This ensured that themes arose from the data, were not presupposed, and that subsequent data could be used to verify the coding structure. Study investigators met periodically to conduct interim analyses and determine when thematic saturation was reached. From the analysis, common themes and provisional hypotheses were generated. Rigour in the collection and analysis of data was promoted by leaving a thorough and transparent audit trail of all analytical and interpretive decisions, keeping a reflexivity journal, and consultation throughout the project with another, experienced qualitative researcher.

## Results

Themes that arose from the interviews fell into 3 broad categories; 1) researchers’ views on the nature of informed consent; 2) researchers’ experiences with obtaining informed consent; and 3) researchers’ views on institutional stakeholders and support available. Themes overlapped between these categories.

### Researchers’ views on the nature of informed consent

#### Definition of informed consent

When asked to define informed consent, researchers identified three key components: information disclosure; understanding; and a decision made voluntarily: “*making sure the person’s aware of what we’re doing, and once they’re aware of it, deciding if they want to participate or not … completely voluntary, no coercion*” [P14].

Researchers also noted that informed consent should be obtained where possible, but that this requirement could be waived under limited circumstances: where privacy could be ensured, and informed consent was impractical to obtain e.g. “*people are dead, you’d have to go back to, you know a million people*” [P08].

Information that researchers thought was important to disclose included: risks associated with participating in the study; the nature of the study; freedom to withdraw from the study; that participants’ decisions whether or not to participate would not affect any other relationship; and contact details of the research team (Supplementary Table 1).

#### Rationale for obtaining informed consent

When researchers were asked why they obtained informed consent, some expressed that it was a legal and regulatory requirement: “*we get it primarily because we have to*” [P08], or because the institute is “*trying to cover themselves*” [P19]. However, the primary rationale was identified as ethical: “*I think there are multiple layers to that, so it is a legal requirement, and ethical requirement, … but also from a personal point of view, I think it is important that when people are volunteering to take part in a trial, that they are provided with enough information*” [P20].

Additionally, researchers noted that honest and informed consent not only improved participants’ compliance and ongoing participation, but also engaged people who were then more likely to enrol. One researcher noted that “*they [potential research participant] usually make their decision as to whether or not to participate based on how you come across to them … if you’ve got the right motives*” [P08] (Supplementary Table 2).

### Researchers’ experiences with obtaining informed consent

“*It’s a negotiation of consent. … rather than having consent as an individual point in time … it’s continually negotiated*” [P13]

Researchers’ accounts of the way they obtained informed consent could be described as involving two distinct phases, referred to here as ‘negotiation’ and ‘operationalisation’. ‘Negotiation’ involved recruitment, information disclosure and all other interactions between a researcher and a potential participant leading to the participants’ decision whether or not to participate. ‘Operationalisation’ was a term used during an interview in this study and describes how a decision made by the participant is recorded.

It should be noted that while the Participant Information and Consent Form (‘PICF’) is often referred to collectively as the ‘consent form’, and is provided to participants as a single document, some researchers saw conceptual and practical differences between the information section (providing information about the study) and the consent component of the form (signing of which indicates consent to participate). Hence, they are presented here as distinct instruments.

#### Negotiating consent with potential participants

Usually, researchers’ initial interactions with potential research participants involved identifying these individuals. This was achieved in a variety of ways, including: referrals from “*somebody who is somehow involved in their care*” [P09]; from advertisements “*on noticeboards, in local newspapers, we speak on the radio*” [P06]; or purposeful sampling from databases.

Once identified, researchers reported using various modes of communication with potential participants, including e-mail messages, facsimile transmissions, telephone calls and face-to-face conversations (Table 2). It was noted that the method of communication used was somewhat driven by the participant. One researcher stated that “… *there’s no single approach that works for everybody*” [P13].

**Table 2:** Views of researchers on modes of communication with participants

	Supporting quotes
**Depends on the study**	“*I think that as risk increases, the information should, the requirement for information increases*” [P18] “*I think the simpler the study and the lower the risk, the smaller the consent should be in the sense of paperwork*” [P01]
**Depends on the participant**	“*some people make it very clear that they’re not actually interested in the nitty gritty, they just want to know big points. Others don’t and that’s where you need to tailor stuff. … the way that I would communicate to a 60 year old Australian man would be different to how I communicate to an 18 year old African woman*” [P12] “*some patients prefer the written word and they can really go away and digest that, and then some patients really um, benefit from a discussion*” [P05]
**Face-to-face interaction**	“*Um, but the verbal is absolutely important, because you have the opportunity to look them in the eyes, and see whether they have this big question mark in their eyes, they don’t quite understand, it’s always better*” [P06]
**Written information form**	“*it’s a matter of us having a more simple form that says “these are the key points that I would like you to know, but here’s a longer document”for example, I think that would be helpful*” [P12] “*if you have too little text, there’s a concern that they’re not, the consent won’t be informed, … but if you have it too verbose, or too, it starts, excluding them from engaging it in a sense*.” [P11] “*Most people … said “no, I don’t need to read it, just tell me about the study.*” [P13]

All researchers reported providing written information to participants: “… *the written is always the minimum*” [P19]. However, despite its prevalence, researchers’ views on the utility of the participant information form varied. It was widely noted that forms could be “*dense … can make participants go ‘oh yeah, just sign it’, without really digesting it*” [P19]. Indeed, researchers reported that study participants often did not read the written information provided to them: “… *nobody reads it because nobody understands. Nobody understands legalese*” [P21]. Some variation was observed, however, with some researchers reporting utility in comprehensive information: “*there’s a reason everything’s there really … even though it’s a bit repetitive*” [P14].

#### The importance of building rapport

To address the fact that few potential research participants read the information form, researchers emphasised the importance of a verbal conversation: “*I think the kind of verbal explanation is good, just to kind of, make it a little bit clearer … it’s more kind of like a compliment to the written word*” [P22]. Researchers mentioned that verbal communication helped “*highlight the really important bits*” [P07]. They also held the view that face-to-face interactions were preferable where possible, as it gave them a chance to “*get a good gist of whether [potential participants] really want to do [the study] or not*” [P01], and also to “*[provide] a better avenue for questions*” [P17], which was seen to promote understanding (Table 2).

Researchers also emphasised that the information discussed with potential participants needed to be tailored to their abilities and interests ([P12], Table 2). The amount and detail of information communicated was also dependent upon the complexity and risk involved in the study: “*the challenge there is that everyone’s idea of what’s rare and serious are different, you know, for some people one in a million is common, for other people that’s very rare*” [P23] (Table 2). When asked how they assessed whether participants understood the information discussed, some interview participants stated that they used ongoing communication: “*we do throughout the session, ask parents if they have any questions in an ongoing way as well*” [P19], and most reported informal methods: “*just about picking up non-verbal cues, is so important*” [P12]. Another researcher stated: “*I get a gut feeling*” [P03]. The time taken to negotiate informed consent also varied, from minutes to months.

Where difficulties arose regarding the participant’s involvement in a study, researchers reported a negotiation of what should be the best outcome for the participant: “*yeah, just like, talking like, just working out if she wanted to continue, what I needed from her if she wanted to continue, just making up a dialogue of like, what’s best for both of us, kind of thing*” [P22].

#### “Operationalising” consent

Researchers reported that they almost always “*operationalise*” [P08] consent with a signed consent form, or in limited cases, consent is implied by the return of a survey. Reasons for always using a signed form included that it would be easier to prove to a third party, that it was necessary for higher risk studies, for administration of data, or that it was convention: “*it’s how it’s always been done. People want written consent*” [P21].

Use of a physical form, however, posed logistical difficulties for some researchers, including the difficulty of distributing the requisite copies to all parties, assembling all the requisite parties physically to sign the form; and the inability to use data when consent forms were lost: “*so you couldn’t use it* [data]*, because there’s no consent form associated with it*” [P13] (Supplementary Table 3).

Very few researchers mentioned the use of verbal consent, and most of those who did reported unfamiliarity with the concept: “*I have drafted a verbal consent form … I still don’t know how it’s going to go … It’s something I’ve never done. But I would like to do*” [P06].

#### The researcher-participant relationship

Researchers indicated that informed consent involves a “*two way street*” [P07], where improved communication between researchers and participants not only benefits the participant, but also allowed researchers to pre-emptively assess whether participants were willing to participate, whether they understood the study, and by extension, whether they would do well in the study (Table 3). This process was reported to be more robust when researchers had better understanding of their participants, so that they could take into account not only inclusion and exclusion criteria, but also vicissitudes in the participants’ lives: “*you can get a bit of a vibe as well if the patient’s able to, to interact with you, so if they’re highly sedated, or looking unwell, you might not approach them*” [P23].

**Table 3:** The value of the researcher-participant relationship

	Quote
**Mutual assessment of suitability**	“*But to understand who this person is, and as much as possible match their care to what they need, it’s also not going to help me or them if you know, it’s not a good match. So for sure, we need to take that into consideration*.” [P09] “*and there’s a kind of two way street, the person needs to understand that so they can commit to whatever the research protocol is, and the researcher has to understand that the person’s participating as a voluntary patient and understands what they’re getting themselves into*” [P07] “*Um, couple of people said to me that they felt that they were under too much stress, and that it was just something else that they didn’t want to do, so I completely backed off of that*” [P09] “*I realised that the patient really wasn’t mentally up to participating in a study, so I didn’t continue*” [P02]

#### Coercion

Most researchers identified that coercion needed to be avoided, and that participants’ susceptibility to coercion was determined by a number of factors, including how a participant is identified, power differentials, poor prognosis, or participants not wanting to disappoint a researcher (Supplementary Table 4). Of note, the majority of participants used the terms “coercion”, “pressure” and “influence” interchangeably.

Researchers noted that protecting participants from coercion was not necessarily achieved by providing more information, rather protection was provided by the study design: *we wouldn’t so much tell them things, we would put protections into the research process to protect them*. *So for example, we would make sure that it was anonymised*” [P13]. Researchers also discussed the need to balance protection of ‘vulnerable’ populations with: “*on the other hand, are you slowing down research that is aiming to … better sort of children’s lives in some way*” [P10].

Researchers expressed frustration towards the perceived increases in paperwork and reviews associated with attempts to address all sources of coercion. There was a sentiment that researchers inherently have good intentions: “*People that are doing this want to do the right thing*” [P12]. One researcher mentioned that: *I think we’re pretty reasonable people, that wouldn’t be going out there to do something to harm somebody*” [P16].

### Researchers’ views on institutional stakeholders and support available

#### Training and guidelines

Few researchers reported receiving formal training on the requirement to obtain informed consent. Of those who did, there were varying reports on the utility of such training, from being a “*tick box exercise*” [P16] with a “*ton of guidelines that I probably will never read through ever*” [P01], to being “*helpful actually, I think it’s good to refresh it*” [P09].

Researchers overwhelmingly reported that their knowledge on informed consent was learnt from on-the-job experience or self-taught. Some also noted that “*if you’re putting consent forms together and writing them, that gives you a really good understanding of … getting stuff across to patients*” [P07]. Researchers who were also members of ethics committees found their committee roles to be a source of training.

When asked about where researchers would seek guidance, many reported that before reading guidelines they would consult peers, research offices, HRECs or the institution website. Researchers stressed the importance of being able to contact a knowledgeable person to ask questions. Researchers reported varying knowledge and familiarity with the National Statement, although most reported at least knowledge that national guidelines exist (Table 4).

**Table 4:** Views of researchers on training and guidelines

	Supporting quotes
**Training**	“*we all have to do ethics training, but again, … I feel like it’s just a tick box exercise. I did it the other day … I just skimmed the case studies, and I didn’t get one MCQ wrong*” [P16] “*Um, well I think having a formal process … is good. I think doing it earlier on, like I think could be part of university training or junior doctor training*” [P07]
**Guidelines**	“*So I know there’s national guidelines, and I did certainly look at them, what they’re called, exactly, national guidelines, or something or another*” [P09] “*the NHMRC code are the ones that we refer to when we need to*” [P15]
**Guidance**	“*I think we’re very fortunate to have this direct contact like you call, someone would answer, and say I’ll take a message, we’ll get back to you, I think they’re doing a fabulous job here*.” [P06] “[*Research office] were so approachable and helpful, so you know, … a couple times I had some questions on things and I could ring them up*” [P09] “*certainly senior researchers will help as well*” [P13] “*If there is something that I didn’t know … I usually go to the contact us page and send an email, or give someone a call*” [P17]

#### The role of HRECs

Researchers’ views on the input of ethics committees varied (Table 5). Some saw HRECs as institutional safeguards, acting as “*a barrier to having inappropriate consent*” [P05] and a source of guidance. While all researchers saw the necessity of ethics review, many also indicated that ethics committees had overly technical and often inconsistent input on information forms and the consent process. This was often a source of frustration. Some researchers characterised the process as a “*necessary evil*” [P14], [P15].

**Table 5:** Views of researchers on HRECs

	Supporting quotes
**Institutional safeguard**	“*Which, which is actually good when the committee comes back and say, this is a question, and you go, “oh, actually I hadn’t thought about that at all”. So makes you think about things and address it*” [P15] “*I just trust the ethics committee here to guide me through it so I don’t make any mistakes, and they would never let me make any mistake*.” [P06]
**Tedious and inconsistent input**	“*So the feedback from ethics committees, so we had seven different ethics committees that we had to go to … and the feedback was different from each of them.*” [P13] “*But it’s made things, ethics committees have made things so [emphasis] hard. So hard. And they’re just getting harder and harder and harder, and the amount of detail that you’ve got to put in, which sometimes, you put the detail in and you think ‘well I don’t know if it’s going to happen exactly like that in every single place that I go to*” [P16] “*I see committees evolve and change from being reasonable to being unreasonable depending on a couple of personalities. And, you know, also committees that go from being chaotic and non-responsive to being very efficient, and, being good*” [P08]
**Necessary**	“*it’s extra paperwork, let’s call it a necessary evil shall we?*” [P15] “*it seemed very tedious at first, it seemed like a lot of work, but I definitely acknowledge and understand the importance of it, because terrible things have been done without it*” [P06] “*I think it’s super necessary, but … you just have to get on top of it. It’s a necessary evil*” [P14]

Some researchers discussed the importance of maintaining ongoing and open communication with their respective ethics officers: ‘*if you haven’t been speaking to your ethics officer, you’re pretty like … it’s not very sensible, because if you want your ethics application to go in, and there be very few things to come back to you, and the way you do that, is to be talking to them first*.’ [P21].

## Discussion

Informed consent is a key concept in human research ethics, yet few studies have investigated researchers’ views on the topic for domestic studies. This study identified that: 1) researchers generally have good awareness of the role of informed consent, its importance and ways to adjust their practice accordingly when obtaining it; and that 2) some researchers felt there was a lack of institutional support on how to obtain informed consent, through lack of training as well as inconsistencies and lack of transparency in HREC requirements.

### Creating the right tools to build rapport

Researchers in our study emphasised that information delivered to potential study participants needed to be tailored to the interests and abilities of each individual. Participant preferences were reported to dictate the modes of communication used, and the complexity of information discussed. To do this effectively, interview participants emphasised the importance of building rapport during a face-to-face conversation to adjust their approaches. Indeed, studies have shown that face-to-face conversations are the best method for improved understanding [18]. The variability of this interaction to individualise the consent process may explain why efforts to protocolise an ideal method for obtaining informed consent have been largely unsuccessful [6, 7].

Despite the view that information delivered must be tailored to the individual, researchers reported that standard written information about the study was always provided, often alongside the consent form. Researchers expressed concern that participants rarely read these documents in detail, and that these were often too long and complicated. Studies show that standardised forms do not result in standardised understanding [18] and that participants’ views on the essential elements of an information form vary widely [30]. Too much information can be overwhelming, and in some cases can impair decision making [31].

Despite the perception that information forms carry limited utility, researchers were reluctant to deviate from using them. Some explained that these forms acted as important reference documents for participants after the informed consent process, and also helped to structure the dialogue, thus reducing the potential for these important informal conversations to be influenced by the biases of individual researchers [31].

Hence, efforts to improve information forms must achieve dual purposes, that they are simplified to be accessible and understandable, but also detailed enough to ensure information delivered is standardised and comprehensive. Rather than merely shortening and simplifying forms [13, 17], efforts should be directed at investigating ways to ensure that these tools are *flexible* and fit for purpose. Indeed, suggestions were made by researchers for the preparation of standard shorter forms, alongside more comprehensive information that can be provided upon request from research participants.

It appears that the ‘best’ way to deliver information to participants, and to ensure participants’ understanding of a study, is to allow researchers some discretion and variation relating to what is discussed. Information forms should be recognised as being an unrealistic reflection of how much information can be, and is actually, conveyed to participants. Studies are required to investigate participants’ perceptions of the utility of written information forms, and whether the shortening of such forms would affect general perception of the legitimacy and safety of studies.

### Researcher as an independent assessor of participation

Researchers described that information provision should be supplemented with both formal and informal assessments of participants’ willingness and rationale for participating. Researchers reported forming an independent view on a participant’s suitability for study inclusion and that if a participant either lacked willingness or had misguided motivations, researchers reported that they would urge participants to reconsider. Researchers did not believe that merely delivering swathes of information was sufficient, but rather that they needed to take care to pre-empt and protect participants from potential sources of undue influence. It appears that the researcher-participant relationship is not an adversarial one, with the researcher vying for more participants and the individual defending themselves by exercising their autonomy to refuse. Researchers saw the informed consent process as a collaborative discussion to ensure that participants made the best decisions for their circumstances. Studies increasingly show that some professional intervention may assist potential participants in making ‘good decisions’ [32]. Researchers identified that an independent assessment of suitability not only benefited the participant, but also saved time and resources for the researcher in the future. This occurred through fewer withdrawals, and improved compliance with study requirements, where participants are aware of, and willingly complete, study requirements.

The literature continues to grapple with balancing the provision of appropriate decision support, while protecting the autonomy of potential participants. The conventional view, enshrined in the National Statement, is that imbalances of power and dependent relationships erode participants’ abilities to make voluntary decisions [1, 9, 19, 20]. Studies are increasingly showing however, that ongoing relationships may enable researchers to provide better decision-making support for potential participants [16]. Researchers in our study were aware of this tension, and some expressed that there existed abstract fear that researchers dogmatically recruit without regard for the safety of the participant. They expressed that this fear was unfounded, and that researchers’ overarching rationales for conducting studies stem from a desire to help others. A more effective conception of the informed consent process may be that an imbalance is not of itself an indication of an invalid decision or one influenced by inappropriate pressure. Rather than a predatory force requiring curtailment, a researcher’s role may be seen as a safeguard that independently assesses whether potential participants are suitable for study inclusion.

### ‘Coercion’ vs ‘undue influence’ – the limits of bioethical discourse

Researchers in our study emphasised the need to avoid coercion and forms of pressure on potential participants when making a decision to participate. Apart from purposes of nomenclature, the unarticulated distinctions between ‘coercion’ and ‘undue pressure’ in Australia have practical ramifications. It is defensible to state that a decision made under the technical definition of ‘coercion’ is invalid. However, the extent to which any influence on a decision is undue, or sufficiently inappropriate to erode the validity of a decision is subject to interpretation and ambiguity. Researchers in this study expressed concern that some measures required by HRECs to ‘avoid coercion’ during the recruitment process would in effect cripple the study. Thus, it is pertinent to derive a consensus statement on the extent to which undue pressures need to be eradicated, if at all. Given the scarcity of guidance provided even in the National Statement, researchers in this study cannot be constructed as apathetic of their ethical obligations. Rather, this misunderstanding highlights that more academic work is required to clarify broad principles on the threshold of unacceptable pressure, and improved education on how those broad principles should be applied in daily practice.

### Support and guidance for researchers

The National Statement in theory sets a national standard on ethical practice for HRECs and researchers. However, awareness and application of these broad principles appear inconsistent. Previous studies have suggested that ethics boards vary in their evaluation of research protocols [33, 34]. This view was also expressed by researchers who participated in our study. Some studies provide possible explanations for variation, including interviews in the US that show ethics review boards struggle with making decisions based on broad guidelines [14], and that Australian HRECs have varying knowledge and use of the National Statement in making decisions [11].

Researchers in our study also reported mixed attitudes towards HRECs, partly as a result of the perceived unpredictability of HREC review. While our study affirms previous work that some researchers view HRECs negatively, reporting impractical and inconsistent demands, we also found contrary views. Those reporting robust communication with HRECs were not only grateful for the guidance HRECs provided but relied on HRECs as institutional safeguards. Poor communication between researchers and HRECs has been identified as a serious problem [12, 35]. While the current study did not aim to, nor was it able to, capture researchers’ practices, we did encounter frustration due to a perceived lack of reasonableness. The National Statement appears to be aware of this phenomenon, and encourages informal in-person discussions between HRECs and researchers to minimise misunderstandings [1]. To promote open dialogue, past studies have recommended researchers attend HREC meetings to advocate for their study protocols and to answer questions in person [10]. Indeed, researchers in our study who were also members of HRECs reported their dual role as a source of training. However, our study shows that the extent to which free communication between researchers and HRECs is promoted appears variable. There was a perception that the overall efficiency and effectiveness of a HREC depended on individuals who were committed to the process.

When ethical concerns arose, researchers reported that they would choose to contact knowledgeable people, before consulting guidelines or rules. An independent, ethics consultative body that has been implemented in parts of the US to address the need for ethical guidance, while alleviating the limited resources of review boards, has shown promising results. Reviews of the board’s function found that it could not only address ethical concerns that arose during the study, which were often outside the purview of the US equivalent of HRECs – institutional review boards (IRBs) – but that it also created a forum for discussion, and a database for challenging and novel cases [36]. This data could be a resource for ethics review boards, and if made accessible to the public, could further help promote open access to, and by extension, trust in, the research enterprise. The feasibility and suitability of implementing an equivalent board in Australia should be further explored.

Most researchers in our study reported that their awareness of informed consent practices arose passively through on-the-job experience, rather than from the use of explicit guidelines or training materials, which is in line with previous research in this area [10]. This form of learning appears to have resulted in a good working knowledge of how to interact with potential participants, especially with regard to the importance of honesty, and of avoiding coercion or undue pressure. However, gaps exist especially in relation to knowledge about regulatory processes surrounding informed consent and ethics review more broadly. Specifically, awareness varied on the National Statement, the types of consent that are permissible, and the role and significance of the information form.

We found that regardless of reported logistical and administrative difficulties, researchers almost invariably believed that written consent was required from participants. Few interview participants were aware of, or had used, verbal or implied consent, despite the National Statement providing that “[c]onsent may be expressed orally, in writing or by some other means” [1]. Interestingly, some researchers interviewed also worked in a clinical capacity, (nurses, doctors, etc.) and were thus familiar with the notion of implied consent in their clinical roles. Reasons for utilising written consent included views that convention dictated it, or that ethics boards required it. A recent study in the US showed that researchers perceived ethics review boards to hold more stringent views on the requirement for consent than they actually did. In particular, researchers underestimated the availability of expedited review for lower risk studies [33]. This may be due to the fact that this type of informal learning magnifies the idiosyncrasies of a working team, and that this would be rarely challenged by external sources. Researchers reported learning about informed consent from creating consent forms from templates that predecessors had used. This results in the preservation of errors which individual researchers may feel powerless to challenge, especially if these become legacy documents and there is lack of support from stakeholder institutions such as HRECs to create new ones. The inconsistencies suggest that practice across different research groups would also vary significantly.

### Study limitations

The main limitation of this study is that semi-structured interviews are not designed to capture behaviour, which will require observational or ethnographic studies. Researchers’ practices discussed here are self-reported. Thus, there is also potential for social desirability bias when describing researchers’ practices to others. Observational research would be timely to reveal the types of information actually disclosed to participants, and deviations of actual practice from perceived practice. However, interviews allowed us to capture researcher views in depth, and helped us to draw richer descriptions of the informed consent process – descriptions that accommodate context in describing phenomena [21, 27]. The use of secondary analysis, a transparent audit trail, and work in iterations, preserved the rigour of our findings, and ample quotes were included in supplementary data to verify interpretations.

Participation in this study was voluntary, which may have resulted in self-selecting bias towards researchers who are more interested in promoting a dialogue relating to informed consent. Another limitation is that the heterogeneous sample may mask some of the issues particular to some types of research, e.g. surveys, which may not be reported in depth here. Future studies should report on difficulties present in particular research types, e.g. research in emergency contexts or with participants with impaired capacity.

## Conclusion

This study highlights the importance of transparent relationships, both between researchers and participants, and between researchers and HRECs to ensure that individuals make good informed decisions about whether or not to participate in research. When the relationship with study participants was reported as more robust, researchers felt that they were able to ensure that participants were not only better informed, but also made better decisions. Where the relationship with HRECs was more robust, researchers were also more likely to view them as institutional safeguards, rather than as bureaucratic hindrances.

While researchers understood the nature and importance of informed consent, they reported a lack in institutional support to navigate regulatory requirements. Researchers reported little awareness of, and support in, implementing more dynamic informed consent procedures, such as verbal informed consent, that was fit for the purposes of their studies.

We propose that establishing an independent consultatory body to deal with issues on informed consent, which would assist in improving communication between researchers and HRECs, provide ethics support and support researchers in designing informed consent procedures.

## Supplementary information

**Additional file 1:.** Interview Guide

**Additional file 2: Supplementary Table 1**: Researchers’ understanding of informed consent. **Supplementary Table 2**: Researchers’ rationale for obtaining informed consent. **Supplementary Table 3**: Researchers views on written consent. **Supplementary Table 4**: The need to avoid coercion

## Data Availability

Anonymised data from the study is available to bona fide researchers on application to the corresponding author.

## Abbreviations

HREC: Human research ethics committee
NHMRC: National Health and Medical Research Council
