# Supplementary material for: Researchers’ views on, and experiences with, the requirement to obtain informed consent in research involving human participants: a qualitative study
Source: BMC Med Ethics. 2020 Oct 2;21:93. doi: 10.1186/s12910-020-00538-7 (PMC7531157; doi:10.1186/s12910-020-00538-7)
Supplement: Supplementary file 1 — Additional file 1:. Interview Guide [file 12910_2020_538_MOESM1_ESM.docx]

Supplementary File 1 – Interview Guide

1. Can you please start by telling me your background (e.g. pharmacy) and how long you have been involved with medical research?

a. How long have you been obtaining consent from potential research study participants?

2. What is informed consent? Can you define it? What is your understanding of informed patient consent?

3. Can you walk me through the process by which you obtain informed consent? Is there any specific approach/tool that you use to do this?

a. What do you think are the most important elements of the consenting process?

b. Who is involved/important in obtaining consent? [Multi-disciplinary team? HREC?]

c. How long does the process normally take?

d. When should consent be obtained?

e. Do you adjust the process by which you obtain consent depending on the type of medical research (for eg. LNR vs interventional) or participant population (eg. Patients with altered consciousness, mental capacity)

f. What determines necessity of consent? Is it required for every study? Who determines this?

g. What happens when a patient revokes consent?

4. Is there any guideline available for clinicians and researchers to assist with obtaining patient consent? (If so, can you describe the guideline? what do you think of them?)

a. Is there any effective communication aid you have come across? (consultation summaries, leaflets/information)

5. Have you encountered any difficulty in obtaining consent from patients?

a. Can you describe these and why you think they occurred?

b. What approaches did you use to overcome them?

c. Is there an approach which you have found that best facilitates the consent process?

6. Do you have any other comment about the process of obtaining informed consent from patients?

7. Do you feel that people you gain consent from largely understand what they are consenting too?
