# Supplementary material for: Researchers’ views on, and experiences with, the requirement to obtain informed consent in research involving human participants: a qualitative study
Source: BMC Med Ethics. 2020 Oct 2;21:93. doi: 10.1186/s12910-020-00538-7 (PMC7531157; doi:10.1186/s12910-020-00538-7)
Supplement: Supplementary file 2 — Additional file 2: Supplementary Table 1: Researchers’ understanding of informed consent. Supplementary Table 2: Researchers’ rationale for obtaining informed consent. Supplementary Table 3: Researchers views on written consent. Supplementary Table 4: The need to avoid coercion [file 12910_2020_538_MOESM2_ESM.docx]

**Supplementary Data**

Supplementary Table 1: Researchers’ understanding of informed consent

|  | **Supporting quotes** |
| --- | --- |
| **Definition of informed consent** | “*the participants or, in some cases, the patient guardian and parents, in the trial need to have, um, sufficient knowledge, so they are willing and active participants in the trial … without coercion, um, and without being misled or under-informed*” [P20] |
| **Information** | “*the core sort of things, to have um what the study’s about, you know, what’s the risk to the participant, you know, what’s the benefit, who to contact, if there’s a problem, I mean that’s that’s probably the main things that you’re looking at*” [P15] |
| **Voluntary** | “*there’s no pressure for them to be part of it or not*” [P07]  *“it's completely voluntary um, withdraw anytime you want*” [P15]  “*So I think you really have a responsibility not to put undue pressure on them*” [P01] |
| **Where consent can be waived** | “*data has to be identi- dei-identified, so that you cannot trace it back to the person. And also, when you present results, it’s aggregated, the results is not, you know, you cannot report on individual. So you, you know, if you make that clear, um, and it’s indeed what you’re doing, then I think in those cases, a waiver is quite reasonable*” [P15]  “*Yeah, I think apart from ones where it’s impractical, people are dead, you’d have to go back to, you know a million people, and get their consent where it’s just not feasible to do it*” [P08] |

Supplementary Table 2: Researchers’ rationale for obtaining informed consent

|  | **Supporting quotes** |
| --- | --- |
| **Ethical: Right to know** | “*it’s like being tricked. If someone is just watching you or observing you, like for what purpose? And for what reason? And if you don’t know that that’s happening, like, is that I don’t know, it’s not really a violation of your privacy, but it’s just you’d rather know that you’re being watched, than just being watched?*” [P16]  “*it's ethical that people understand what it is that they're participating in*” [P09] |
| **Ethical: Respect for volunteers** | “*You're benefiting from them being involved and there should be a mutual understanding of that benefit, then that they’re not being taken advantage of*” [P01]  “*you can’t be doing things to people and people’s samples without their permission*” [P07]  “*But when you’re involved, enrolling someone in research, they’re going out of their way to do something they don’t have to, and it’s to help me*” [P12] |
| **Trust and compliance** | “*the compliance of the patient over your intervention is improved with a better consent process*” [P01]  “*if we want families to come back again, I think that makes it more likely that they’re going to do that as well, yeah if they feel like they know what they’re getting in for*” [P10]  “*they do better if it's of their own volition and there's no coercion at all*” [P05] |

Supplementary Table 3: Researchers views on written consent

|  | **Supporting quotes** |
| --- | --- |
| **Consent form use** | “*For every, for every study that requires informed consent, yes*” [P20] |
| **Reasons for using written consent** | “*There seems to be a bit of… something signed is sort of better than [verbal consent]. I don’t know where that sort of comes from, presumably ethics committees*.” [P08]  “*I think you definitely need a consent form for things that are complex*” [P13]  “*Patients had been consented over the phone, and one of those consent forms went missing, and it was very very difficult to trace back and find the consent*” [P02] |
| **Difficulties of a physical form** | “*If you’re doing research remotely, it’s almost impossible to do that, because they sign it and then send it back to you, so, are you meant to then sign, co-sign it, take a copy and then send them back a copy?*” [P13]  “*Participants don't come in frequently to sign*” [P03]  “*I can’t even do a blood pressure … not being able to do, vital signs, and histories, and paperwork, is a big problem*.” [P02] |

Supplementary Table 4: The need to avoid coercion

|  | **Supporting quotes** |
| --- | --- |
| **Sources of coercion** | “*I find the link between consent and coercion, or recruitment and consent, is um, I think that relationship is important*,” [P18]  “*They think that need to do it because they’re at the hospital and the hospital’s done a lot for them*” [P01]  “*patients often talk about their relationship with the treating team … how it’s very personable and personal … I need to respect that, as part of my process of obtaining consent, and really stress that it doesn’t make a difference to their care*.” [P09] |
| **How to protect against coercion** | “*I think if you are a lay person, going into a trial, you’re not likely to have come across those sorts of things before, so the onus is on the people doing the trial, and the ethics committee that review the trial, to ensure that, you know, appropriate safeguards are in place*” [P20]  “*if you have a sufficiently rigorous, or independent relationship between recruitment and consent, then I think you can provide a uh, reduced risk of coercion, and have a appropriate informed consent*” [P18] |
| **Researchers wish to do good** | “*I think we're pretty reasonable people, that wouldn't be going out there to do something to harm somebody*” [P16]  “*I don’t think it’s deliberate, but a lot of times, because you’re so invested in your research, you might not see the side effects of it*” [P15] |
| **Protection versus progress** | “*the ‘v’ word* [‘vulnerable’] *for me is one of the most abused words in the ethics field … researching that population* [is] *very different from the community. When really, they’re still people, they’ve got two arms, two legs, you know, they can make decisions, they can say yes, they can say no*.” [P08] |
